# Fluorescent CXCR4 targeting peptide as alternative for antibody staining in Ewing sarcoma

**DOI:** 10.1186/s12885-017-3352-z

**Published:** 2017-05-26

**Authors:** Laurens G. L. Sand, Tessa Buckle, Fijs W. B. van Leeuwen, Willem E. Corver, Alwine B. Kruisselbrink, Aart G. Jochemsen, Pancras C. W. Hogendoorn, Károly Szuhai

**Affiliations:** 1 Department of Pathology, Leiden University Medical Center, Leiden, The Netherlands; 2 Interventional Molecular Imaging Laboratory, Department of Radiology, Leiden, The Netherlands; 3 Division of Molecular Pathology, The Netherlands Cancer Institute, Amsterdam, The Netherlands; 4 Department of Molecular Cell Biology, Leiden University Medical Center, Leiden, Postbus 9600 2300 RC, The Netherlands

**Keywords:** Chemokines, Bone tumor, Sarcoma, Molecular imaging, Flow cytometry, Peptides, Live cell imaging

## Abstract

**Background:**

Ewing sarcoma is an aggressive, highly metastatic primary bone and soft tissue tumor most frequently occurring in the bone of young adolescents. Patients, especially those diagnosed with a metastatic disease, have a poor overall survival. Chemokine receptor CXCR4 has a key pro-tumorigenic role in the tumor microenvironment of Ewing sarcoma and has been suggested to be involved in the increased metastatic propensity. Earlier studies on CXCR4 protein expression in Ewing sarcoma yielded contradictory results when compared to CXCR4 RNA expression studies. Previously, we demonstrated that CXCR4 expression could be detected in vivo using the fluorescently tagged CXCR4-specific peptide MSAP-Ac-TZ14011. Therefore, we studied the membranous CXCR4 expression in Ewing sarcoma cell lines using MSAP-Ac-TZ14011.

**Methods:**

The CXCR4 membrane expression levels were studied in EWS cell lines by flow cytometry using the hybrid peptide MSAP-Ac-TZ14011 and were correlated to CXCR4 RNA expression levels. The measurements were compared to levels detected using the CXCR4 antibody ab2074 under various cell preparation conditions. In addition, the staining patterns were analyzed by confocal fluorescence microscopy over time.

**Results:**

The hybrid peptide MSAP-Ac-TZ14011 levels showed a strong and better correlation of CXCR4 membrane expression with the CXCR4 RNA expression levels than observed with the anti-CXCR4 antibody ab2074. With the hybrid peptide MSAP-Ac-TZ14011 using live cell confocal microscopy CXCR4 membrane staining and internalization was detected and the signal intensity correlated well with CXCR4 mRNA expression levels.

**Conclusions:**

The fluorescently labeled CXCR4 targeting peptide-based method provides a reliable alternative to antibody staining to study the CXCR4 membrane expression in live cells using either flow cytometry or live cell fluorescence microscopy. The fluorescently tagged CXCR4 targeting peptide could enable in vivo detection of CXCR4 expression in Ewing sarcoma which may help to stratify cases for anti-CXCR4 therapy.

**Electronic supplementary material:**

The online version of this article (doi:10.1186/s12885-017-3352-z) contains supplementary material, which is available to authorized users.

## Background

The tumor microenvironment (TME) has a key role in metastasis, angiogenesis and tumor growth [1–3]. Chemokines are important signaling molecules in the TME [4]. The chemokine signaling axis that is involved in all main processes of the TME is the Chemokine (C-X-C Motif) Ligand 12 (CXCL12), also known as stromal derived factor 1, and the Chemokine (C-X-C Motif) Receptor 4 (CXCR4) axis [5, 6]. CXCR4 expression has been associated with metastasis and tumor progression in various tumor types, including Ewing sarcoma (EWS) [7–10]. EWS is an aggressive primary malignant neoplasm occurring dominantly in bone in children and young adolescents [11]. Primary extraskeletal soft tissue presentation is more frequent in adults [12]. The five year overall survival in patients with a localized disease at diagnosis is 70% but drops to 10–30% when patients have a metastatic disease at diagnosis or a recurrence [13, 14]. The fact that approximately 25% of the patients present metastases at the time of diagnosis potentially implies an important role for CXCR4, which is the highest expressed chemokine receptor in EWS [8]. Expression studies at RNA and protein level revealed that high CXCR4 expression levels were associated with a decreased survival in EWS patients [8, 9]. However, when the expression of CXCR4 in metastases was analyzed controversial results were obtained with “high” RNA expression levels and “low” or absent protein expression. A plausible explanation for this discrepancy could be related to the used anti-CXCR4 antibody used in this study which recognizes an N-terminal epitope. Furthermore, immunohistochemistry staining patterns of CXCR4, which is a membrane receptor, were also reported in other studies in the nucleus and cytoplasm [15–17].

Reliable detection of CXCR4 could help to clarify the role of CXCR4 in tumors. CXCR4 has been the target for the development of a variety of imaging agents [18]. Of these agents the fluorescently labeled, derivatives of the antagonistic peptide (T140) proved to be of value [19–23]. Moreover, in a more recent study such a T140-analogue has been successfully applied to longitudinally monitor the CXCR4 expression in a ductal carcinoma *in situ* breast cancer model [24]. Therefore, we reasoned that the same peptide analogue could also help to clarify the CXCR4 expression levels at the cell membrane in EWS. To investigate this, we used the T140 analogue MSAP-Ac-TZ14011 to discriminate between CXCR4 “high” and CXCR4 “low” EWS cell lines using live cell imaging and flow cytometry. In addition, we evaluated the effect of variation in the flow cytometry preparation protocol on the detected fluorescence. The flow cytometry measurements were compared to the *CXCR4* RNA expression levels of the used cell lines.

## Methods

### Cell culture

EWS cell lines were obtained from multiple sources: L1062 was established in-house [25]; A673 (ATCC® CRL-1598™) and MDA-MB-468 (ATCC® HTB-132™) were obtained from the American Type Culture Collection; 6647 was kindly provided by Dr. Timothy Triche (CHLA, Los Angeles, CA, USA) and TC32, VH64, IARC-EW3, RM82 and IARC-EW7 were obtained from the EuroBoNET consortium collection (Institute of Pathology, University Medical Center, Düsseldorf, Germany) [26]. All EWS cell lines were cultured in Iscove’s Modified Dulbecco’s Medium (IMDM) with GlutaMAX supplement, supplemented with 10% heat-inactivated fetal calf serum (FCS) (all from Life Technologies). The B-lineage acute lymphoblastic leukemia (B-ALL) cell line “Leiden-ALL-HP” was kindly provided by the Department of Hematology, Leiden University Medical Center, Leiden, The Netherlands and was cultured as described earlier [27]. MDA-MB-231 X4, a human breast cancer cell line which stably overexpresses a GFP-tagged version of the human CXCR4 receptor [28], was kindly provided by Gary Luker (University of Michigan Medical School, MI, USA) and cultured in DMEM supplemented with 10% heat-inactivated FCS (all Life Technologies, Bleiswijk, The Netherlands). This cell line was used as control during the whole study. Regular Mycoplasma DNA Q-PCR screening [29] and Cell-ID STR typing using PowerPlex 1.2 (Promega, Leiden, The Netherlands) were conducted as quality control.

### Fluorescent peptide

This study made use of the previously reported hybrid peptide MSAP-Ac-TZ14011, consists of the CXCR4 targeting peptide Ac-TZ14011. A DTPA chelate capable to bind a radioactive Indium and a Cy5.5 fluorophore, which enables both single-photon emission computed tomography (SPECT) detection and fluorescence imaging. The dissociation constant (K_d_) and specificity of the peptide were described earlier [30].

### Confocal imaging

Cells were plated on a glass bottom culture dish (MatTek Corporation, Ashland, Ma, USA) 24 h before imaging. Imaging of cells was performed upon incubation with MSAP-Ac-TZ14011 (0.27 μM) at standard culture conditions. Binding and internalization was assessed in real-time in MDA-MB-231 X4; images were collected every 2 min for 3 h. EWS cell lines TC32 and IARC-EW7 were imaged prior to, directly after addition of MSAP-Ac-TZ14011 to the culture medium (*T* = 0) and 3 h after incubation with MSAP-Ac-TZ14011 (*T* = 3). Prior to imaging at *T* = 3 cells were washed, lysosomes were stained using lysotracker DND-26 (0.5 μM) and the nucleus was stained with Hoechst (1:2500 1 mg/ml) (both Life Technologies) to discriminate between cytoplasm and nucleus. Imaging was performed on a SP5 microscope with a HCX PL APO 63.0 × 1.40 OIL lens (Leica, Eindhoven, The Netherlands) at the microscope facility of the Department of Molecular Cell Biology, Leiden University Medical Center. Used excitation lasers and emission detection ranges are in Additional file 1: Table S1. Images were collected and evaluated using the LASAF software (Leica).

### Flow cytometry

Cells were dissociated with trypsin (Life Technologies) and resuspended in 10% fetal calf serum (FCS), IMDM media. Subsequently, cells were washed and incubated in a blocking buffer PBS 5% BSA (PBA) for 30 min at 4 °C. Afterwards cells were incubated with MSAP-Ac-TZ14011 (0.27 μM) for 1 h in PBA at 4 °C and washed 3 times with PBA at 4 °C. Propidium iodide (1 μM, Sigma-Aldrich GmbH, Steinheim am Albuch, Germany) was added 30 min prior to flow cytometry measurement to separate dead cells from vital cells. For the comparison with ab2074 (1:50, Abcam, Cambridge, United Kingdom), the antibody used in the EWS study [9], live cells were prepared for flow cytometry analysis as described by Pelekanos et al. [31] using the secondary Goat anti Rabbit antibody conjugated to Alexa 647 (1:200, ThermoFisher Scientific, Breda, Netherlands). This specific protocol was used since it did not fix the cells and would therefore be comparable to the other used protocols. The used filters and lasers to measure the fluorescent signals are listed in Additional file 2: Table S2.

To investigate the effect of cell dissociation procedure on the intactness of the receptor and binding of the ligand, cells were handled using three methods: either Trypsin (0.025% without EDTA) or TripLE (all from Life Technologies) or 10 mM EDTA (Sigma-Aldrich GmbH) dissolved in PBS, pH 7.4.

To investigate the effect of fixation on the flow cytometric measurements, methanol fixation was performed. The MSAP-Ac-TZ14011 staining was followed by washing with PBS at 4 °C and fixation with 100% methanol (−20 °C) by adding the fixative drop-wise. Cells were then stored in 95% methanol for 20 min at −20 °C, followed by washing with ice cold PBS. All measurements were performed on a LSRII flow cytometer (BD Biosciences, San Jose, CA, USA). Data files containing information from at least 10.000 live (propidium iodide (PI) negative) single cell events were analyzed using WinList 8.0 based (Verity software House, Topsham, ME, USA). Fluorescent intensity was indicated by relative fluorescent intensity (RFI). Cells stained with PI and the secondary antibody Alexa647 were used for background.

#### RNA isolation, RT-Q-PCR analysis and Fluidigm

RNA expression of CXCR4 was determined as previously described [10]. In brief, total RNA was isolated using TRIzol Reagent for cDNA generation. RT-Q-PCR was performed using the Fluidigm BioMark HD system (Fluidigm, San Francisco, CA, USA). Samples were measured in duplicates and analyzed using BioMark software, delivered with the HD system.

#### Western blot

Cell lysates were prepared using Giordano buffer (50 mM Tris-HCl, pH 7.4, 0.1% Triton X-100, 250 mM NaCl, 5 mM EDTA) supplemented with phosphatase and protease inhibitors (Sigma-Aldrich). After separation of the protein on SDS-PAGE, blotting and blocking with 10% low fat skimmed milk, membranes were incubated with Ab2074, NBP1–76867 and UMB2 (all were Rabbit antibodies and were used in 1:200 dilution, Novus Biologicals, Littleton, CO, USA) or anti-Vinculin antibody (loading control). Goat anti-rabbit horseradish peroxidase-conjugated (1:10,000, Jackson Laboratories, Bar Harbor, ME, USA) was used secondary antibody and detected with ECL.

### Statistical analysis

Linear regression analysis was performed by using Graphpad Prism 6 (Graphpad Software Inc. La Jolla, CA, USA).

## Results

### Flow cytometry using MSAP-ac-TZ14011 on live EWS cells

**Fig. 1:**
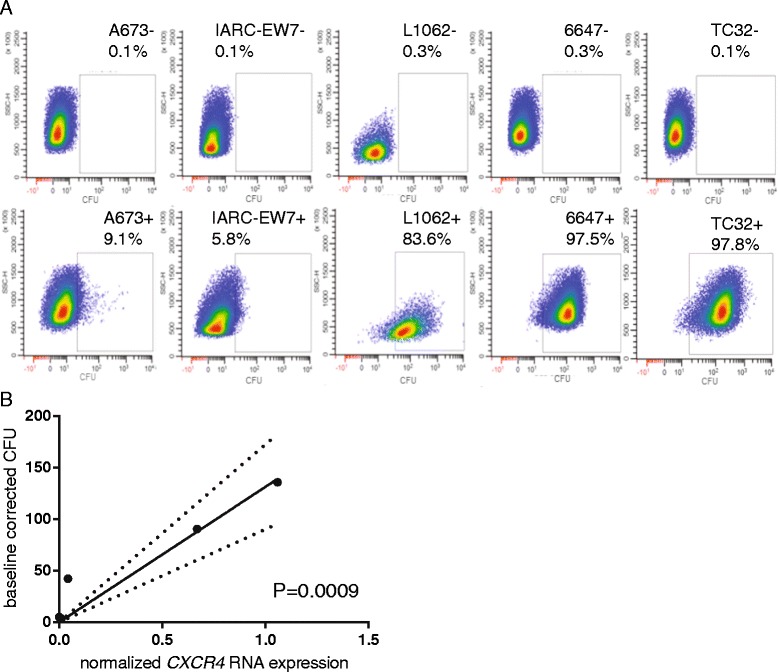
Semi-quantitative detection of MSAP-Ac-TZ14011 in EWS cell lines correlated significantly with *CXCR4* RNA expression levels. **a** Cells of the EWS cell lines A673, IARC-EW7, L1062, 6647 and TC32 were harvested and stained without (*upper graphs*) or with (*lower graphs*) MSAP-Ac-TZ14011. Fluorescence was detected at 710–40 nm. The calculated percentage of positive cells is indicated in each panel. **b** The baseline corrected geometric mean cytometry fluorescence intensities (GFI) detected after MSAP-Ac-TZ14011 staining of the in (**a**) described EWS cell lines were correlated to the previous determined *CXCR4* RNA expression levels (x-axis). Linear regression analysis demonstrated a significant correlation between the by MSAP-Ac-TZ14011 detected CXCR4 levels and RNA expression levels (*P*-value and 95% certainty borders are displaced). Both figures are representatives (*n* = 3)

CXCR4 cell membrane expression levels detected by MSAP-Ac-TZ14011 of five EWS cell lines with varying *CXCR4* RNA expression levels (IARC-EW7, A673, L1062, 6647 and TC32) [10] were quantified by flow cytometry. Within the previously tested panel of 20 EWS cell lines, A673 and IARC-EW7 demonstrated very low CXCR4 RNA expression levels, L1062 demonstrated a moderate CXCR4 RNA expression level, and 6647 and TC32 demonstrated high CXCR4 RNA expression levels. In IARC-EW7 and A673 almost no CXCR4 cell membrane expression was detected (>10%). In TC32 and 6647 CXCR4 cell membrane expression was observed in almost all cells (>90%) (Fig. 1a). Within the population, varying detection levels were observed with standard deviations ranging from 160.4 to 873.36 GFI, although no clear separate populations were identified (Fig. 1a). The variation in fluorescence within the cell lines were consistent to earlier observations [32]. The baseline corrected geometric means of the measured MSAP-Ac-TZ14011 levels were correlated to the earlier obtained *CXCR4* RNA expression levels [10]. A significant linear correlation (*P* = 0.0009) was obtained between these two conditions in various cell lines (Fig. 1b).

### Flow cytometric comparison of MSAP-ac-TZ14011 with ab2074 and validation of the cell-preparation effects on these staining

**Fig. 2:**
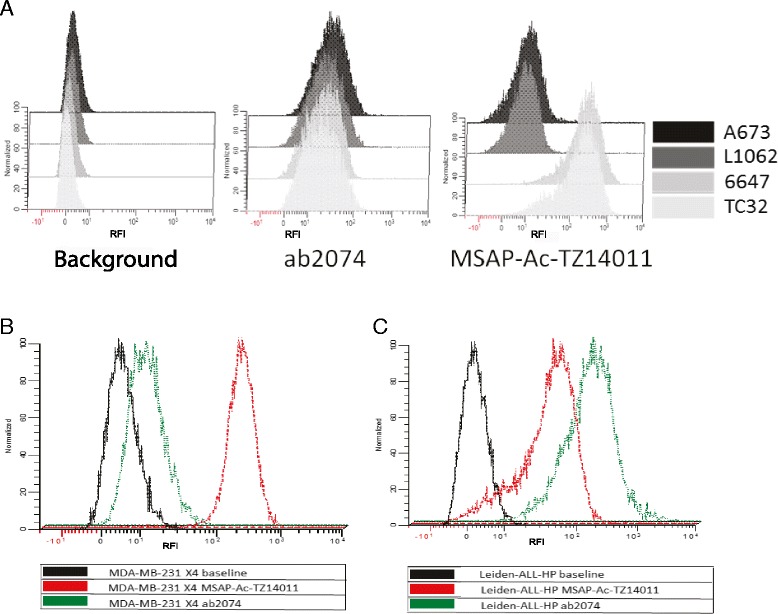
Fluorescence comparison between ab2074 and MSAP-Ac-TZ14011 staining. **a** EWS cell lines TC32 (*white*), 6647 (*light gray*) (high *CXCR4* RNA expression), L1062 (*dark gray*) and A673 (*black*) (low *CXCR4* RNA expression) were similar prepared for the ab2074 and MSAP-Ac-TZ14011 staining following the protocol described by Pelekanos et al. [31]. The ab2074 did not demonstrate a difference in CXCR4 detection between high and low *CXCR4* RNA expressing cell lines where MSAP-Ac-TZ14011 did. Representative figures (*n* = 3) are demonstrated. **b** MDA-MB-231 X4 and **c** “Leiden-ALL-HP” cells were prepared and stained similar to the EWS cell lines. In MDA-MB-231 X4 the same difference between ab2074 (*green*) and MSAP-Ac-TZ14011 (*red*) staining was demonstrated but in “Leiden-ALL-HP” a higher expression was detected by ab2074 than MSAP-Ac-TZ14011 (both *n* = 1). The normalized number of counts is demonstrated

The MSAP-Ac-TZ14011 fluorescence levels were compared to the levels obtained with the anti-CXCR4 antibody ab2074. In contrast to the findings with MSAP-Ac-TZ14011 peptide, anti-CXCR4 ab2074 antibody staining did not demonstrate any difference between the studied EWS cell lines (Fig. 2a). In addition, in the MDA-MB-231 X4 cells a lower CXCR4 signal was detected when using ab2074 compared to MSAP-Ac-TZ14011 (Fig. 2b). As a positive control cell line of different origin growing in suspension the “Leiden-ALL-HP” cell line was used. In this cell line the ab2074 antibody CXCR4 detected signals were higher than the MSAP-Ac-TZ14011 levels (Fig. 2c).

The difference in signal intensity levels of the anti-CXCR4 antibody ab2074 detection might be caused by cell preparation as the Leiden-ALL-HP grown in suspension and EWS cells are harvested by enzymatic dissociation to obtain cell suspension. Therefore different cell dissociations methods were compared using regular trypsin (enzyme digestion) TrpLE (recombinant enzyme used in stem cell research to treat trypsin sensitive cells) and EDTA alone (non-enzymatic treatment). None of the harvesting methods (EDTA, Trypsin and TrpLE) influenced any of the staining methods (Additional file 3: Figure S1). In addition, we observed that fixation with methanol after MSAP-Ac-TZ14011 incubation increased the detected fluorescence, both in high and low CXCR4 expressing cell lines (Additional file 4: Fig. S2).

### Live cell imaging of CXCR4 by MSAP-ac-TZ14011 in EWS cells

**Fig. 3:**
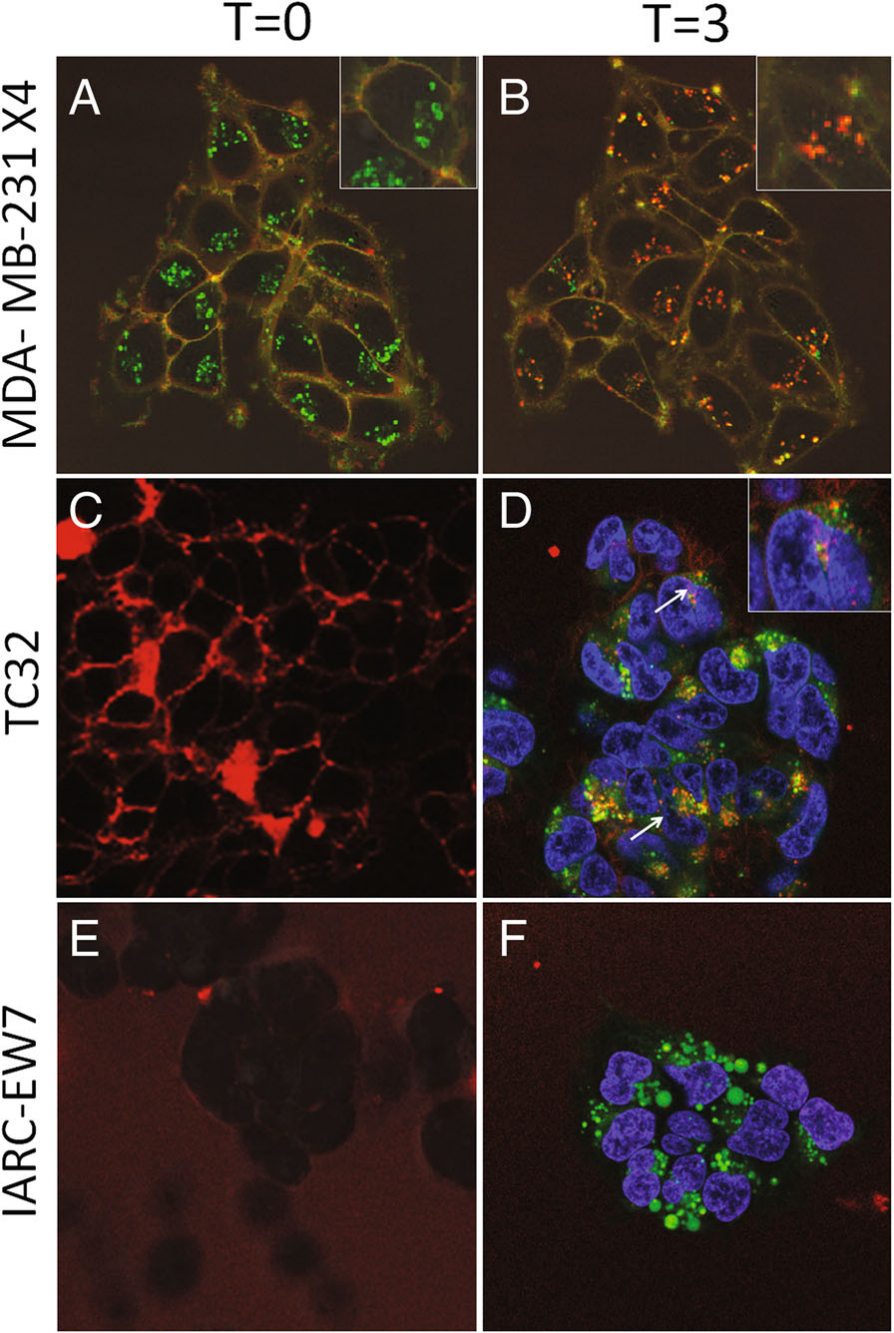
Live cell validation of MSAP-Ac-TZ14011 staining in EWS cells. **a**, **b** MDA-MB-231 X4 containing transfected CXCR4-GFP (*green*) and EWS cell lines (**c**, **d**) TC32 and (**e**, **f**) IARC-EW7 were imaged by live cell confocal microscopy directly after incubation with MSAP-Ac-TZ14011 (*red*) (*T* = 0) and after 3 h incubation (*T* = 3). At *T* = 0 MDA-MB-231 X4 and TC32 demonstrated a membrane staining where no staining was observed in IARC-EW7. IARC-EW7 and TC32 were half an hour prior to imaging incubated with lysotracker DND-26 (*green*). Hoechst staining (*blue*) was used to stain the nucleus. All images were taken using a 63× objective. In the MDA-MB-231 × 4 cell line at *T* = 3 intracellular MSAP-Ac-TZ14011 signal was detected that was co-localized with CXCR4-GFP signal (panel **b** and *inset*). In TC32 cell the lysotracker DND-26 signal was co-localized with the MSAP-Ac-TZ14011 signal (**d**, *arrow* and *inset*)


EWS cell lines TC32 and IARC-EW7 were further investigated by live cell imaging. TC32 and IARC-EW7 had, respectively, “high” and “low” *CXCR4* RNA expression levels [10] and “high” and “low” CXCR4 levels detected by MSAP-Ac-TZ14011. As control for estimation of the MSAP-Ac-TZ14011 incubation period MDA-MB-231 X4, in which overexpressed CXCR4-GFP is located at the membrane and in the cytoplasm, was imaged over time (Fig. 3a, b and Additional file 5: Movie S1). Directly after addition of MSAP-Ac-TZ14011 (*T* = 0) all membrane expressed CXCR4-GFP overlapped with MSAP-Ac-TZ14011 as shown in Fig. 3a in orange. The intracellular CXCR4-GFP signal was not overlapping with MSAP-Ac-TZ14011 signal. Over time MSAP-Ac-TZ14011 was internalized with CXCR4-GFP and after 3 h almost all CXCR4-GFP present was bound by MSAP-Ac-TZ14011 (*T* = 3) (Fig. 3b and Additional file 5: Movie S1, which contains a time-laps recording). This includes the CXCR4-GFP located intracellularly. TC32, a high *CXCR4* mRNA expressing EWS cell line, demonstrated at *T* = 0 a membranous MSAP-Ac-TZ14011 staining similar to that demonstrated by MDA-MB-231 X4 (Fig. 3c). After 3 h incubation internalized MSAP-Ac-TZ14011, like in MDA-MB-231 X4, was observed in TC32 (Fig. 3d). The signal partly overlapped with the lysotracker DND-26 signal, indicating that a part CXCR4-MSAP-Ac-TZ14011 complex was directed towards the lysosomes. The low *CXCR4* expressing EWS cell line IARC-EW7 cells showed neither cell membrane staining at *T* = 0 nor cytoplasmic staining at *T* = 3 of MSAP-Ac-TZ14011, suggesting no binding and internalization of CXCR4 was observed (Fig. 2e, f).

Additional file 5: Movie S1. live cell imaging of MDA-MB-231 X4 cells. Live cell imaging of MDA-MB-231 X4 over 3 h after the addition of MSAP-Ac-TZ14011. Every 2 min the distribution of MSAP-Ac-TZ14011 (red) and GFP (green) were imaged, demonstrating an overlap (yellow) of the two signals at the membrane and internalization of both signals from the membrane into the cytoplasm. All images over a time of 3 h were combined using LASAF software (Leica). (MOV 1766 kb)


## Discussion

In this study we have demonstrated that a CXCR4 targeting fluorescent T140 analogue, MSAP-Ac-TZ14011 tracer peptide, can be used as an alternative for antibodies to determine the CXCR4 cell membrane expression levels in EWS cell lines and that the binding of MSAP-Ac-TZ14011 is not significantly influenced by the used cell dissociation method. The measured levels of cell bound MSAP-Ac-TZ14011, and thereby indirectly the measured CXCR4 levels, were correlated to *CXCR4* mRNA expression and compared with CXCR4 cell membrane levels detected by antibody staining. As the CXCR4 signaling pathway have a stimulating role in the main processes of the TME in many tumor types, CXCR4 could be a candidate biomarker and a potential therapeutic target. [5, 7]. Moreover, treatment with CXCR4 antagonist T140 and analogues like Ac-TZ14011 inhibited tumor growth [33]. In EWS, however, although RNA expression has been reported, protein expression in metastases was absent in paraffin embedded material using immunohistochemistry [8, 9]. CXCR4 consists of multiple isoforms with varying N-terminal ends of which one, CXCR4–2, is dominantly expressed [34]. Both the N- and C-terminal ends of CXCR4 contain many potential post-translational modification sites and changes at these sites may influence antibody recognition, potentially explaining the various staining patterns observed in earlier studies [35–37]. In addition, CXCR4 protein expression analysis by Western blotting with N-terminal and C-terminal specific antibodies revealed inconclusive staining patterns (Additional file 6: Fig. S3). As the T140 binding site does not contain any reported post-translational modifications and *in situ* modeling suggests MSAP-Ac-TZ14011 binds at the same site, this detection could be a better alternative to detect all forms of the receptor [18, 23, 35, 38]. The detected MSAP-Ac-TZ14011 signals correlated linearly with the earlier obtained RNA expression levels in EWS cell lines and co-localize with CXCR4-GFP expressed in MDA-MB-231 X4 (Figs. 1b and 2a, b). In addition, the detected MSAP-Ac-TZ14011 levels using flow cytometry corresponded to the observations during live cell imaging of the cells (Figs. 1a and 2c, e). Limitations of this method are that cells should be stained alive with MSAP-Ac-TZ14011 and not fixed with methanol prior to staining.

The observed internalization of the MSAP-Ac-TZ14011 in TC32 and MDA-MB-231 X4 confirms previously reported internalization of Ac-TZ14011-FITC [22]. The CXCR4-GFP overlap with MSAP-Ac-TZ14011 during CXCR4 internalization in MDA-MB-231 X4 and the overlap of MSAP-Ac-TZ14011 with the lysotracker DND-26 in TC32 support the suggestion that upon CXCR4 binding the peptide-CXCR4 complex is internalized. This peptide therefore can be used to detect intracellular located CXCR4 in cells when incubated over a longer period at standard culture conditions. In addition, Ac-TZ14011-based analogues have been studied in in vivo breast-tumor models based on immune uncompromised mice, and the efficacy of this approach has been shown in multiple publications [20, 24, 39, 40]. One CXCR4-targeting imaging agent has even been studied in a recent clinical trial, indicating the suitability of it to identify tumor cells in patients [41]. In the preclinical setting the in vivo biodistribution of Ac-TZ14011 analogues was shown to be identical to the distribution in immune-deficient nude mice bearing low CXCR expressing control tumors, with exemption of the uptake levels in the tumor. Evaluation of immune cells in the CXCR4 positive tumors revealed the presence of minor amounts of immune cells in the tumor (1.0–1.3%), which did not influence the visualization of the tumor [24]. This indicates that effective tumor detection would still be possible, even when a CXCR4 positive stromal cell infiltrates are present. Based on this, we propose that the in vivo detection of CXCR4 expressing tumors in EWS patients might well be possible.

CXCR4 is involved in metastasis and increased CXCR4 RNA expression levels were measured in both metastasis derived cell lines compared to non-metastasis derived cell lines and metastases compared to localized tumors [8]. In addition, factors inhibiting CXCR4 activation can be used to identify high risk patients [10]. The detected MSAP-Ac-TZ14011 levels in EWS cell lines positively correlated with the CXCR4 RNA expression levels and the MSAP-Ac-TZ14011 signal overlapped with the CXCR4-GFP membrane signal. When assuming the MSAP-Ac-TZ14011 fluorescence level is correlated to the CXCR4 cell membrane level, metastasis might have a higher CXCR4 cell membrane expression than localized tumors. Such a positive correlation between the migration/invasiveness of a cell line and the CXCR4 cell membrane expression has been observed both in EWS and breast cancer cell lines [24, 32]. However, clinical data on the cell membrane expression of CXCR4 and its relation with tumor invasiveness is still lacking. Using this method might enable determination of CXCR4 cell membrane expression in patients. This would help to stratify patients for alternative therapies, like anti-CXCR4 therapy and might serve as prognostic marker for EWS patients [9].

## Conclusions

In conclusion, staining with the fluorescent CXCR4 targeting peptide MSAP-Ac-TZ14011, by using live cell imaging and flow cytometry, resulted in fluorescence levels that corresponded to the CXCR4 RNA expression levels of the used EWS cell lines. This peptide-based method was appropriate for studying qualitatively and semi-quantitatively CXCR4 cell membrane expression in live cells in EWS and other cell types and might be well suited for future in vitro and in vivo CXCR4 studies.

## Additional files

Additional file 1: Table S1. Live cell imaging excitation and emission settings. This table present the imaging parameters with life cell imaging. (DOCX 15 kb)

Additional file 2: Table S2. Flow cytometry laser and filters. (DOCX 14 kb)

Additional file 3: Fig. S1. Validation to show the effect of cell harvesting procedure on detected MSAP-Ac-TZ14011 and ab2074 signal intensities. The effect of 10 μM EDTA (green), TripLE (red) and 0.025% trypsin (blue) treatment on the fluorescence of ab2074 (*) and MSAP-Ac-TZ14011 staining were tested on (A) MDA-MB-231 X4, (B) TC32 and (C) A673. As representative baseline (black), the result of TripLE treatment without additional staining was used. No significant difference in fluorescence was observed. The Y-axis represents the normalized number of cell counts (*n* = 1). (EPS 1732 kb)

Additional file 4: Fig. S2. Influence of methanol fixation after MSAP-Ac-TZ14011 staining. EWS cell line A673 and MDA-MB-231 X4 were stained with MSAP-Ac-TZ14011 and subsequently were (black) or were not (gray) fixed with methanol. Methanol fixation of the cells lead to increased fluorescence levels. The Y-axis represents the normalized number of cell counts (*n* = 1). (EPS 1315 kb)

Additional file 6: Fig. S3. Western blot analysis of CXCR4 protein expression with N- and C-terminal directed antibodies. Western blot analysis of CXCr4 protein expression in high CXCR4 expressing Ewing sarcoma cell line IARC-EW3, medium CXCR4 expressing breast cancer cell line MDA-MB-468 and low CXCR4 expressing Ewing cell line IARC-EW7 (supplementary results [10]) using two N-terminal antibodies (Ab2074 and NBP1–76867) and one C-terminal antibody (UMB2). Theoretical molecular weight of CXCR4–2 is 39 kDa. No conclusive results could be obtained with any of these antibodies. (EPS 959 kb)

## Abbreviations

EWS: Ewing sarcoma
FCS: Fetal calf serum
IMDM: Iscove’s Modified Dulbecco’s Medium
PBA: PBS 5% BSA
PBS: Phosphate-buffered saline
PI: Propidium iodide
RFI: Relative fluorescence intensity
SPECT: Single-photon emission computed tomography
TME: Tumor microenvironment
