# Supplementary material for: Fluorescent CXCR4 targeting peptide as alternative for antibody staining in Ewing sarcoma
Source: BMC Cancer. 2017 May 26;17:383. doi: 10.1186/s12885-017-3352-z (PMC5446759; doi:10.1186/s12885-017-3352-z)
Supplement: Supplementary file 2 — Live cell imaging excitation and emission settings. This table present the imaging parameters with life cell imaging. (DOCX 15 kb) [file 12885_2017_3352_MOESM1_ESM.docx]

**Table S1: Live cell imaging excitation and emission settings**

| Signal | Laser | Excitation filter | Detection emission |
| --- | --- | --- | --- |
| DND-26 lysotracker | Argon | 496 | 510-530 |
| Cy5.5 | Argon | 633 | 650-700 |
| Hoechst | Diode | 405 | 415-505 |
| GFP | Argon | 488 | 500-550 |
