# Supplementary material for: Fluorescent CXCR4 targeting peptide as alternative for antibody staining in Ewing sarcoma
Source: BMC Cancer. 2017 May 26;17:383. doi: 10.1186/s12885-017-3352-z (PMC5446759; doi:10.1186/s12885-017-3352-z)
Supplement: Supplementary file 3 — Flow cytometry laser and filters. (DOCX 14 kb) [file 12885_2017_3352_MOESM2_ESM.docx]

**Table S2: Flow cytometry laser and filters**

| Signal | Laser | Filter |
| --- | --- | --- |
| Cy5.5 | Red | 710-40 |
| Alexa647 | Red | 660-20 |
| PI | Blue | 695-40 |
| GFP | Blue | 530-30 |
